# Unlocking the potential of artificial intelligence in improving learning achievement in blended learning: a meta-analysis

**DOI:** 10.3389/fpsyg.2025.1691414

**Published:** 2025-10-31

**Authors:** Jiajun Wu, Ahmed Tlili, Soheil Salha, Daria Mizza, Mohammed Saqr, Sonsoles López-Pernas, Ronghuai Huang

**Affiliations:** ^1^Faculty of Education, City University of Macau, Taipa, Macau SAR, China; ^2^Smart Learning Institute of Beijing Normal University, Beijing, China; ^3^An-Najah National University, Nablus, Palestine; ^4^Department of Educational Studies, American University in Cairo, New Cairo, Egypt; ^5^School of Computing, University of Eastern Finland, Kuopio, Finland

**Keywords:** blended learning, hybrid learning, artificial intelligence, smart learning, learning achievement, learning performance, meta-analysis, quantitative evidence

## Abstract

**Introduction:**

While several studies investigated the effect of blended learning on students’ learning achievement, scant information exists on whether using artificial intelligence (AI) in blended learning could further contribute to the obtained effect.

**Methods:**

To effectively address the challenges and opportunities presented by blended learning and AI, the present study conducts a meta-analysis to systematically examine the impact of AI-enhanced blended learning on students’ learning achievement, considering the significant role of multiple variables in shaping this achievement, including the type of AI technology, instruction duration, research design, and sample size as well as across different educational levels and subject areas. Specifically, 21 studies (*N* = 2,873 participants) were meta-analyzed.

**Results:**

The obtained results revealed that AI has a medium effect (*g* = 0.5) on students’ learning achievement in blended learning. Particularly, personalized systems in blended learning had the highest effect (i.e., large) compared to chatbots and intelligent tutoring systems. Finally, it is seen that the educational context (grade level and educational subject), as well as the experiment type (research design, intervention duration, and sample size), moderate the effect of AI on students’ learning achievement in blended learning.

**Discussion:**

The findings of this study can help researchers and practitioners better understand the effects of AI in blended learning, thereby contributing to a better design of teaching and learning experiences accordingly.

## 1 Introduction

The concept of Blended Learning (BL) has evolved significantly over the past few decades, building upon traditional distance education and face-to-face instruction (Mizza and Rubio, 2020). As technology has advanced, so too has the potential of BL to enhance the learning experience. BL seamlessly integrates the benefits of both online and in-person learning (Mizza and Rubio, 2020). It provides opportunities for personalized feedback, collaborative activities, and asynchronous communication, making it particularly suitable for all students, with diverse learning profiles and those in remote locations (Garrison and Kanuka, 2004; Graham and Dziuban, 2007; Watson, 2008; Wanner and Palmer, 2015).

With the advent of the Fourth Industrial Revolution, Artificial Intelligence (AI) has emerged as a transformative technology in educational technology ecosystems (Holmes et al., 2019). AI-powered adaptive learning systems and intelligent tutoring systems are now enabling educators to create more sophisticated and personalized BL environments through advanced algorithmic approaches. These advanced capabilities are offered through machine learning-driven content recommendation engines, dynamic assessment adaptation mechanisms, and intelligent learner support systems. In doing so, AI demonstrated remarkable pedagogical personalization capabilities that were hardly realizable at the scale before (Kumar et al., 2024).

Nevertheless, while AI-enhanced BL holds promise in fostering personalization and enhancing students’ learning outcomes, research is needed to fully understand its potential impact (Kumar et al., 2024). A significant challenge in evaluating BL practices has been the lack of consistent, comprehensive evaluation criteria (Yan and Chen, 2021; Zhang et al., 2022). Han (2023) has highlighted the importance of considering multiple factors when assessing BL’s impact on student learning outcomes. More robust literature synthesis is crucial to avoid inflating outcomes or obfuscating results. The present study addresses this gap through a comprehensive meta-analysis that considers a wide range of variables, including AI technology type, intervention duration, research design, sample size, and educational contexts across different grade levels and subject areas. The findings will discuss effective integration strategies to help educators make informed, equitable decisions about AI-enhanced BL solutions.

## 2 Literature review

### 2.1 Blended learning and AI impact

While no standard definition of impact exists, this study defines impact as the measurable outcomes of a student’s achievement in both the online and in-person components of BL (Spanjers et al., 2015). Positively influencing students’ academic achievement is a fundamental objective shared by educators, institutions, and societies at large. Nevertheless, research on the impact of BL has thus far shown mixed results, emphasizing the need to understand what makes teaching approaches effective and how they work in different contexts.

Since the early 2000s, particularly in higher education in the United States, there has been evidence of a positive impact when comparing BL approaches to traditional learning methods. Numerous studies have documented improvements across various educational dimensions. Researchers have linked BL to enhanced student achievement and increased motivation (Vaughan, 2014), better support mechanisms (Lim et al., 2019), improved access to comprehensive learning materials (Kim et al., 2014), and greater overall engagement and achievement (Owston et al., 2013; Bernard et al., 2014; Halverson et al., 2014; Spanjers et al., 2015; Boelens et al., 2017; Asarta and Schmidt, 2020; Rasheed et al., 2020). This trend extends across both higher and secondary education, offering personalized learning experiences and broader educational opportunities (Picciano, 2012; Hilliard, 2015).

However, other studies have found no significant contribution of BL to student achievement and test scores compared to traditional learning environments (Means et al., 2013, Müller et al., 2018). These conflicting findings can be attributed to a range of complex and interconnected factors. BL design features, including technology quality, online tools, and face-to-face support, interact closely with student characteristics such as technological proficiency (Alfadda and Mahdi, 2021), individual attitudes, and self-regulation capabilities. Contextual factors further complicate the assessment, with instructors’ expertise, subject matter, and specific course goals playing crucial roles in determining learning outcomes.

With the emergence of AI, many researchers sought to explore its potential and embed its capabilities in different applications. Due to the recency and the rapid development of AI, researchers sought to answer the question of impact by exploring the potential of AI to enhance BL environments. A systematic review by Park and Doo (2024) examining AI applications in BL from January 2007 to October 2023 revealed several promising developments in educational technology. AI-powered tools and platforms may create flexible and personalized learning experiences. These AI technologies demonstrate remarkable capabilities in tailoring content to individual needs, providing personalized instruction and scaffolding (Liao and Wu, 2022; Phillips et al., 2020), adapting assessments to student achievement, and offering personalized guidance and feedback (Jovanović et al., 2017; Liao and Wu, 2022). Nevertheless, despite these promising developments, the research on AI’s impact on BL remains inconclusive. While some studies show potential benefits, AI integration does not consistently lead to improved learning outcomes across different educational levels. Challenges persist, including data dependency, the ongoing need for human expertise, and the lack of standardized measurement tools. Future investigations should focus on systematically measuring AI’s effect on student learning achievement and understanding the intricate interplay of factors influencing its effectiveness.

### 2.2 Previous meta-analyses on blended learning

To address the challenges of comprehensively evaluating the integration of BL and AI, which impedes systematic understanding of their effectiveness and impact on educational outcomes, meta-analysis offers a powerful analytical approach. Meta-analyses systematically aggregate data from multiple studies, providing valuable insights that inform the development of new or revised BL criteria and contribute to establishing more robust and standardized evaluation methods (Cohn and Becker, 2003).

Unlike individual studies that may yield varying results due to differences in methodology, sample size, or other contextual factors, meta-analysis can reconcile these discrepancies by aggregating data, thereby increasing sample size and statistical power. This methodological approach makes it more likely to detect significant effects and help identify moderating factors that influence BL effectiveness, such as the specific blend of face-to-face and online instruction, student characteristics, or instructor expertise.

Several meta-analyses have examined the effectiveness of BL. While earlier research supported the perspective that BL can result in better learning outcomes for higher education students (Means et al., 2010, 2013; Bernard et al., 2014), recent meta-analyses have yielded mixed results, highlighting the complexities of implementing BL in various educational settings. Vo et al. (2017) conducted a cross-regional meta-analysis with 51 studies in higher education and they found that the effect of BL on student achievement is small.

A more recent study by Yu et al. (2022) analyzed 30 peer-reviewed studies encompassing 70 effect sizes, exploring BL’s impact on student outcomes and attitudes. The findings indicated that BL significantly outperforms traditional instruction, with a medium effect size. Students in BL environments demonstrated higher academic achievement and more positive learning attitudes. However, the researchers emphasized that BL effectiveness varies depending on the implementation model, student characteristics, and the quality of instructional materials and technologies.

Another significant meta-analysis by Xu L. et al. (2022) and Xu Z. et al. (2022) explored the potential of self-regulated learning (SRL) interventions in online and BL environments. This research examined the efficacy of self-regulated learning on academic achievement through the moderators of article type, subject type, learning context, educational level, SRL strategy, SRL phase, SRL scaffolds, and intensity and duration of intervention. The study revealed a moderate effect of SRL intervention on academic achievement in online and blended environments.

Cao's (2023) meta-analysis provided an additional perspective by evaluating BL effectiveness across different countries. While BL generally demonstrated a positive moderate impact on student achievement, attitudes, and achievement, engagement levels varied significantly.

Despite the limited number of meta-analyses on blended learning, the findings from these studies vary to some extent. While the effect size reported by Vo et al. (2017) is small, it was found to be medium in Yu et al. (2022), Xu L. et al. (2022) and Xu Z. et al. (2022). Additionally, the focus of each meta-analysis differed. For example, Cao (2023) concentrated on comparing effect sizes across several countries, while Vo et al. (2017) focused solely on higher education, and Xu L. et al. (2022) and Xu Z. et al. (2022) centered on self-regulated learning as the main factor. It is clear that the initial meta-analyses identified several gaps to be explored, such as the various applications of AI in BL and the different educational levels. Additionally, the existing meta-analyses further highlight the complexity of BL on learning achievement and the need for context-specific strategies, particularly when integrating AI. They emphasize the importance of ongoing research to understand the many factors that contribute to creating effective BL environments.

## 3 Research gap and study objectives

As mentioned in the previous section, while several meta-analyses have examined the effectiveness of BL, no research, to the best of the authors’ knowledge, has specifically investigated the impact of AI on student achievement within BL environments. However, while there are some systematic reviews of AI on blended learning (Park and Doo, 2024; Al-Maroof et al., 2022), all of them were qualitative and did not provide quantitative evidence on how AI would impact students’ learning achievement in blended learning. Therefore, more rigorous quantitative studies are needed to establish a definitive causal link between AI and improved students’ learning achievement. In other words, fragmented evidence was found related to the impact of AI on learning achievement in blended learning. Thus, to better understand this effect, a meta-analysis and synthesis is needed. Meta-analyses are effective in this context as they synthesize results from multiple studies and sources to provide an overall effect size, offering a more comprehensive understanding of AI effect. To address this research gap, the present study quantitatively measures the effect of AI on learning achievement through a systematic review and meta-analysis.

In addition, it is seen that the effect of AI in education is moderated by several variables, including educational subject and level (Vo et al., 2017), intervention duration, geographical distribution, and learning context (Xu L. et al., 2022; Xu Z. et al., 2022). Therefore, to fully understand the effectiveness of AI in BL environments, it is essential to consider various factors, such as the specific implementation of the BL model, the characteristics of the students, the used instruction and technologies, and the broader educational context. Thus, the impact of AI in BL needs to be further unpacked to better understand its effect on learning achievement. To do so, the present study takes one step forward and investigates what might moderate the effect of AI in blended learning. Specifically, it explores the impact of its integration whose magnitude is influenced by a wide range of critical variables. Therefore, the research question is: ‘To what extent do AI-enhanced BL environments improve student learning achievement, and what critical variables influence the magnitude of these effects?’

To address this research question, the present study identifies certain factors that were not adequately explored in previous research and must be prioritized in this meta-analysis to contribute to a more comprehensive understanding. It expands the scope to examine BL by considering specific types of applications across various grade levels and different subject areas. Additionally, this study focuses on specific factors such as the intervention duration, the research design, and the sample size. Accordingly, the following two main research questions were further developed and guided this research:

*RQ1*. What is the overall effect of AI on student learning achievement in blended learning environments?

*RQ2*. How does the effectiveness of AI in blended learning vary across different moderators, including grade level, educational subject, instruction duration, study design, and sample size?

The findings of this study can contribute to the ongoing debate related to the effectiveness of AI in education generally and in blended learning particularly. It advances understanding of AI’s role in pedagogical frameworks and the different variables to be considered when developing AI-based interventions.

## 4 Methodology

### 4.1 Search and data retrieval

A search was performed in the electronic databases of Science Direct, IEEE Xplore, Taylor & Francis, Scopus, and Web of Science, as these databases are familiar in the field of AI and blended learning, and include several of the most important journals. The search strings were adapted from several AI and blended learning reviews in the literature (e.g., Park and Doo, 2024), and are as follows: (Artificial intelligence substring) AND (blended learning) AND (education substring), where:

- *Artificial intelligence substring*: “artificial intelligence” OR AI OR “machine intelligence” “OR “machine learning” OR “natural language processing” OR “deep learning” OR robotic.
- *Blended learning substring*: “blended learning” OR “hybrid learning” OR “flipped learning” OR “integrated learning” OR “multi-method learning.”
- *Education substring*: “learning achievement” OR “learning performance” OR “academic achievement” OR “academic performance.”

The included articles were peer-reviewed to ensure the quality of the meta-analysis. Moreover, the search period was set starting from 2011, since this year was considered as the year where AI applications became more mature and AI assisted technology was booming (Wang et al., 2023).

**Figure 1:**
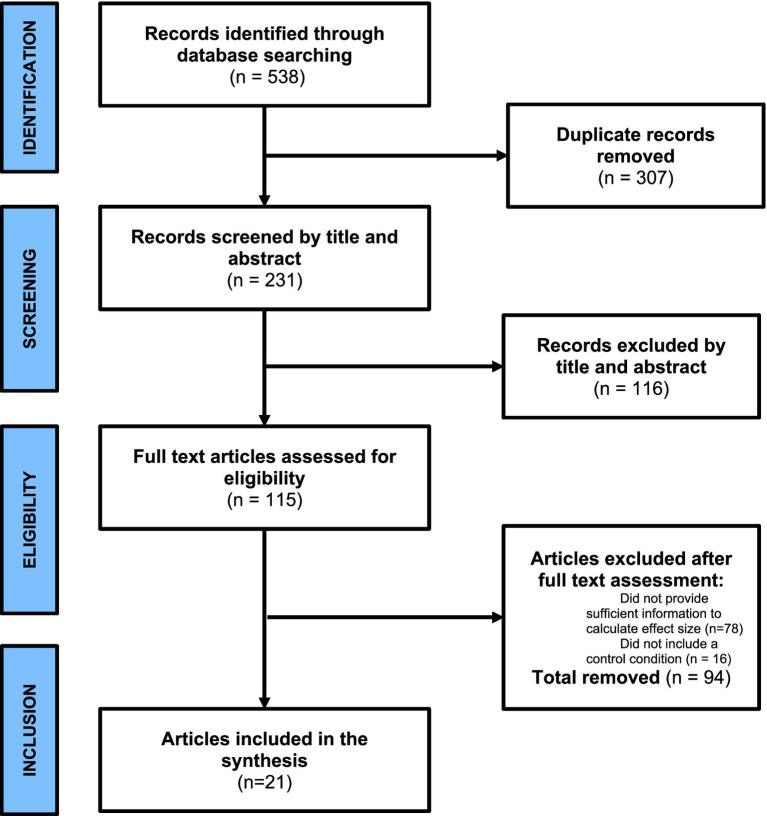
PRISMA chart.

The last search was performed on June 01, 2024, and the overall process yielded 538 potential studies. A 307 potential studies were removed due to duplication, then the inclusion/exclusion criteria were applied. A study was included if it: (1) was in English; (2) was an empirical research; (3) used AI for blended learning; (4) was not qualitative or review research since these studies do not have the needed statistical data (e.g., mean effect sizes; standard errors and confidence intervals; and the samples sizes) to conduct a meta-analysis; (5) provided sufficient information (e.g., mean, and standard error) to compute the effect size; or (6) included a control condition. It should be noted that no study was excluded based on the used AI application and the obtained set of AI applications in this study were the result of the aforementioned inclusion/exclusion criteria. Finally, 21 studies (2,873 participants in total) were considered for this meta-analysis. There were 16 studies followed true experimental design and 5 studies followed quasi experimental design. Figure 1 presents the data selection process.

### 4.2 Meta-analysis

The software of Comprehensive Meta-Analysis V.4 was used to conduct this meta-analysis. Hedges’ *g* was used to calculate the effect sizes (Hedges, 1981). The motivation behind using Hedges’ *g* instead of Cohen’s d effect size is to provide a less biased estimate of the effect size (Tlili et al., 2023). Hedges’ *g* incorporates a small sample correction factor that reduces the upward bias that can occur in Cohen’s d with limited data (Hedges and Olkin, 1985). Nineteen studies followed the pretest–posttest-control (PPC) research design. In the current research design, students are assigned to experimental and control interventions and are evaluated before and after the intervention (i.e., the learning process). As stated by Morris (2008), the PPC design provides reliable and precise values of effect sizes and minimizes the threats to internal validity. The other two papers followed the design of posttest only with control (POWC), where participants are assigned to experimental and control interventions and evaluated just after the intervention (i.e., learning process).

Four methods were used to assess publication bias. The first method is the trim-and-fill with the focus of identifying publication bias by means of a funnel plot wherein the papers are represented by dots. It is assumed that there is no publication bias when the dots are distributed on both sides of a vertical line representing the average effect size (Borenstein et al., 2010). The second method was Rosenthal’s (1979) fail-safe number which aims to bring the meta-analytic mean effect size down to a statistically insignificant level. A fail-safe number larger than 5 *k* + 10 (where *k* is the original number of studies included in the meta-analysis) is robust. It suggests that publication bias is unlikely to significantly affect the overall results (Borenstein et al., 2021). The third method was Egger’s regression test where a significant intercept suggests publication bias. The fourth method was *p*-curve analysis, which assesses whether the distribution of statistically significant *p*-values in the included studies demonstrates evidential value or is indicative of selective reporting practices (Simonsohn et al., 2014).

## 5 Results

### 5.1 Effect of AI in blended learning

**Figure 2:**
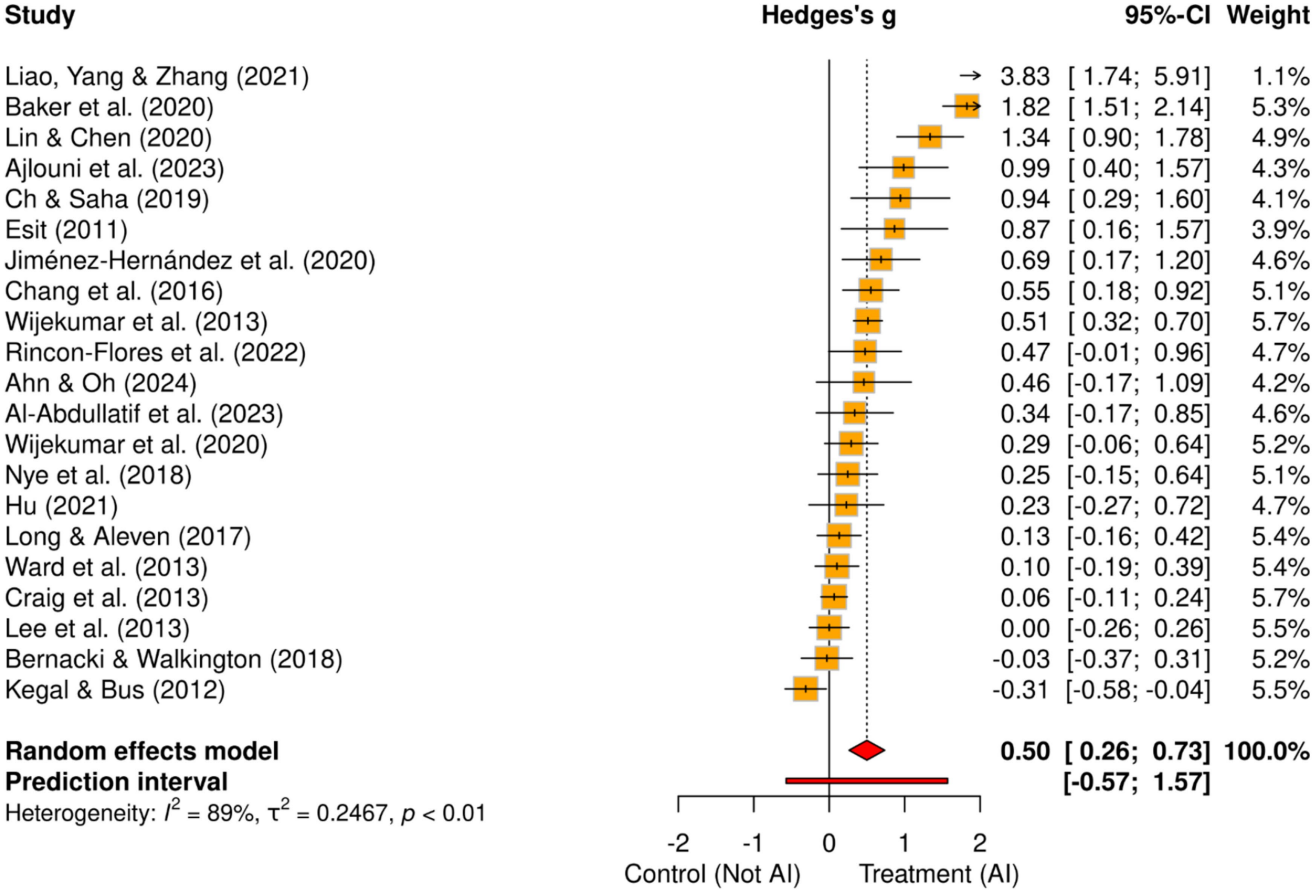
Forest plot.

The overall pooled effect size of the 21 studies shows a medium effect size (Hedges’ *g* = 0.50), with a 95% confidence interval of [0.27, 0.74] (Table 1). The *z*-value is 4.17, and the effect is statistically significant (*p* = 0.001). There was substantial variability across the included studies manifested in high heterogeneity values (*I*^2^ = 88.97, *τ*^2^ = 0.25). As such the prediction interval (Figure 2) ranged from −0.57 to 1.57 indicating that future replication of the positive results of the meta-analysis may be unwarranted.

**Table 1:** Effect of AI on blended learning environment.

Analysis	*k*	*g*	95% CI	*Z*	*p*	*I^2^*	*τ* ^2^	ES interpretation
Overall	21	0.50	[0.27, 0.74]	4.17	0.001***	88.97	0.25	Medium
ChatBot	1	0.34	[−0.17, 0.84]	1.32	0.188	0	0	Small
ITS	16	0.42	[0.15, 0.69]	3.09	0.002**	90.19	0.24	Small-medium
Personalized systems	4	0.88	[0.48, 1.27]	4.33	0.001***	60.89	0.10	Large

*k*, number of studies; *g*, Hedges’ g effect size (ES); CI, confidence interval; *Z*, *Z* value for Hedges’ *g*, *p*, *p* values of Hedges’ *g*, *I*^2^ and *τ*^2^ are measures of effect size variability. ***p < 0.001.

Looking at the differences by the type of AI implemented (Table 1), it is evident that studies that used a personalized AI system (*k* = 4) reported a large effect size (Hedges’ *g* = 0.88), which was statistically significant (*p* = 0.001, 95% CI [0.48, 1.27]). The heterogeneity was moderate (*I*^2^ = 60.89, *τ*^2^ = 0.10) and lower compared to other categories, indicating more consistent results for the personalized approach. Intelligent Tutoring Systems (ITS) (*k* = 16) studies reported a small-to-medium and statistically significant effect size (Hedges’ *g* = 0.42, 95% CI [0.15, 0.69], *p* = 0.002), although heterogeneity was high (*I*^2^ = 90.19, *τ*^2^ = 0.24). Lastly, only one study used a chatbot system and reported a small effect size (Hedges’ *g* = 0.34), which was non-significant (95% CI [−0.17, 0.84], *p* = 0.188). The *I*^2^ statistic showed that 88.97% of variance resulted from between-study factors, implying that other variables might moderate the effect size of AI in blended learning.

### 5.2 Moderating effect of grade level

There were marked variations in grade levels (Table 2) where the highest effect sizes were obtained in higher education and the lowest in early childhood studies. In that, studies in higher education (*k* = 9) showed a large effect size (Hedges’ *g* = 0.7, 95% CI [0.03, 1.02], *p* = 0.001), although with considerable heterogeneity (*I*^2^ = 70.41%, *τ*^2^ = 0.15). Primary education (*k* = 2) followed, with a medium effect size (Hedge’s *g* = 0.55), although the results are not statistically significant (95% CI [−0.14, 1.25], *p* = 0.12) and showed considerable heterogeneity. Secondary education studies (*k* = 9) yielded a small-to-medium effect size (Hedges’ *g* = 0.40) which was statistically significant, although the studies presented very high heterogeneity (*I*^2^ = 93.07%, *τ*^2^ = 0.26). Lastly, the analysis of early childhood education included only one study which reported a small negative effect size (Hedges’ *g* = −0.31). The negative effect was statistically significant (95% [−0.58, −0.04], *p* = 0.02).

**Table 2:** Effect of AI on blended across various grade levels on learning achievement.

Grade level	*k*	*g*	95% CI	*Z*	*p*	*I^2^*	*τ* ^2^	ES interpretation
Early childhood	1	−0.31	[−0.58, −0.04]	−2.25	0.02*	0	0	Small
Primary education	2	0.55	[−0.14, 1.25]	1.56	0.12	66.87	0.17	Medium
Secondary education	9	0.40	[0.05, 0.75]	2.25	0.03*	93.07	0.26	Small-medium
Higher education	9	0.70	[0.03, 1.02]	4.32	0.001***	70.41	0.15	Large

*k*, number of studies; *g*, Hedges’ *g* effect size; CI, confidence interval; *Z*, *Z* value for Hedges’ *g*, *p*, *p* values of Hedges’ *g*, *I*^2^ and *τ*^2^ are measures of effect size variability. ***p < 0.001, *p < 0.05.

### 5.3 Moderating effect of educational subject

The meta-analysis sheds light on the varied impact of AI-enhanced blended learning across educational subject areas (Table 3). Notably, in teacher training, AI demonstrated a substantial positive effect, with a very large effect size (Hedges’s *g* = 0.99), although only one study was conducted in this subject. Similarly, a large effect size was observed in the five studies in ICT and Engineering (Hedges’s *g* = 0.88). However, the results in other subjects were not decisive. In Languages, although there was a moderate effect size of 0.56, the lack of statistical significance and high variability among studies (*I*^2^ = 95.26%) suggest unclear and inconsistent results. Mathematics presented a modest outcome, with a negligible effect size of 0.11. In Science and Social Sciences, AI had a moderate effect size of 0.44 with statistically insignificant effect size and moderate heterogeneity (*I*^2^ = 67.75%).

**Table 3:** Effect of AI on blended learning across various educational subject areas on learning achievement.

Educational subject	*k*	*g*	95% CI	*Z*	*p*	*I^2^*	τ^2^	ES interpretation
ICT and engineering	5	0.88	[0.31, 1.45]	3.00	0.003**	80.39	0.30	Large
Languages	6	0.56	[−0.05, 1.17]	1.80	0.07	95.26	0.54	Medium
Mathematics	6	0.11	[−0.03, 0.26]	1.56	0.09	28.80	0.01	Negligible
Science and social sciences	3	0.44	[−0.02, 0.90]	1.86	0.06	67.75	0.11	Small-medium
Teacher training	1	0.99	[0.41, 1.56]	3.34	0.001***	0	0	Very large

*k*, number of studies; *g*, Hedges’ g effect size; CI, confidence interval; *Z*, *Z* value for Hedges’ *g*, *p*, *p* values of Hedges’ *g*, *I*^2^ and *τ*^2^ are measures of effect size variability. ***p < 0.001, *p < 0.05.

### 5.4 Moderating effect of instruction duration

The effectiveness of AI-enhanced blended learning varies significantly depending on the duration of the instruction (Table 4). The strongest impact was seen in short-term duration (*k* = 3, 1 week to 1 month), with a very large effect size of 1.14, indicating AI’s substantial immediate influence. This effect was consistent across studies. In longer durations (*k* = 14, between 1 month and 1 semester), the effect size was moderate 0.53, significant but with very high variability across studies (*I*^2^ = 90.97). In long-term durations (*k* = 4, 1 semester to 1 year), the effect size was almost negligible and non-significant 0.08. These variations may be explained—at least partially—by the immediacy effect where closer time to assessment may result in a more positive effect as students are more likely to retain what they have learned.

**Table 4:** Effect of AI on blended with different instruction durations on learning achievement.

Instruction duration	*k*	*g*	95% CI	*Z*	*p*	*I^2^*	*τ* ^2^	ES interpretation
1 week ≤ duration < 1 month	3	1.14	[0.82, 1.46]	6.97	0.001***	0	0	Very large
1 month ≤ duration < 1 semester	14	0.53	[0.22, 0.84]	3.33	0.001***	90.97	0.29	Medium
1 semester ≤ duration < 1 year	4	0.08	[−0.08, 0.23]	0.99	0.32	0	0	Negligible

*k*, number of studies; *g*, Hedges’ *g* effect size; CI, confidence interval; *Z*, *Z* value for Hedges’ *g*, *p*, *p* values of Hedges’ *g*, *I*^2^ and *τ*^2^ are measures of effect size variability. ***p < 0.001, *p < 0.05.

### 5.5 Moderating effect of study design

Regarding study design, studies using an experimental design reported a statistically significant medium effect size of 0.52 (Table 5). This effect was inconsistent across the studies giving rise to high levels of heterogeneity (*I*^2^ = 91.35%). In contrast, studies employing a quasi-experimental design reported a slightly lower and statistically significant effect size of 0.42. Yet, the effect was more consistent across the studies with heterogeneity measures of (*I*^2^ = 49.16%).

**Table 5:** Effect of AI on blended learning in different study designs on learning achievement.

Study design	*k*	*g*	95% CI	*Z*	*p*	*I^2^*	*τ* ^2^	ES interpretation
Quasi	5	0.42	[0.12, 0.71]	2.76	0.006**	49.16	0.05	Small-medium
True	16	0.52	[0.24, 0.81]	3.56	0.001***	91.35	0.29	Medium

*k*, number of studies; *g*, Hedges’ *g* effect size; CI, confidence interval; *Z*, *Z* value for Hedges’ *g*, *p*, *p* values of Hedges’ *g*, *I*^2^ and *τ*^2^ are measures of effect size variability. *p < 0.001.

### 5.6 Moderating effect of sample size

Table 6 reveals that studies with smaller sample sizes (≤250 participants) showed a moderate effect size of 0.57, which was statistically significant (*p* = 0.001). On the other hand, studies with larger sample sizes (>250 participants) showed a much smaller effect size of 0.24, which was not statistically significant (*p* = 0.121). Both groups show a very high heterogeneity (I^2^ > 80%).

**Table 6:** Effect of AI on blended learning in different sample size on learning achievement.

Sample size	*k*	*g*	95% CI	*Z*	*p*	*I^2^*	τ^2^	ES interpretation
Small (≤250)	18	0.57	[0.27, 0.88]	3.67	0.001***	89.67	0.37	Medium
Large (>250)	3	0.24	[−0.06, 0.54]	1.55	0.121	83.75	0.06	Small

*k*, number of studies; *g*, Hedges’ *g* effect size; CI, confidence interval; *Z*, *Z* value for Hedges’ *g*, *p*, *p* values of Hedges’ *g*, *I*^2^ and *τ*^2^ are measures of effect size variability. *p < 0.001.

### 5.7 Meta-regression

By and large, the results of meta-regression (Table 7) reflect the results of the subgroup analysis. Regarding the grade level, the analysis shows that AI is less effective in Early Childhood (EC) and Primary Education (PE), with both having negative coefficients of −0.45. The impact on the overall effect size was statistically significant (*p* = 0.04) in Early Childhood, though negative. In contrast, Secondary Education (SE) shows a significant positive coefficient of 0.87 (*p* = 0.001), suggesting that AI interventions may be more likely to be effective for older students. Regarding educational subjects, the field of teacher training shows a positive coefficient of 0.80, with marginal significance (*p* = 0.05). On the contrary, a significant negative impact was reported in Mathematics, with a coefficient of −0.38 (*p* = 0.001). This suggests that AI applications or tools may be less ready for Mathematics education. Regarding instruction durations, shorter durations (1 week to 1 month) show a non-significant positive effect (coefficient = 0.14), implying that the benefits of AI are more apparent in the short term. In contrast, the analysis shows that longer durations (1 semester to 1 year) are associated with a significantly negative coefficient of −1.28 (*p* = 0.001), suggesting that the positive effects of AI be lost or—better said—not retained over time. Put another way, AI might show strong initial results, but sustaining these benefits over longer periods can be challenging—which is not only specific to AI education. Regarding research design, compared to experimental design, quasi-experimental designs show a significantly lower effect size, as indicated by a negative coefficient of −0.58 (*p* = 0.02). Similarly, compared with studies with smaller sizes, studies with larger sample sizes (>250 participants) have a significantly negative coefficient of −1.15 (*p* = 0.001).

**Table 7:** Meta-regression results for the learning achievement of response from grade level, educational subject, instruction duration, study design and sample size.

Model	Variable	Coef.	SE	95% lower	95% upper	*z*-value	2-sided *p* value	
Intercept		0.77	0.17	0.43	1.10	4.53	0.001	
Grade level	1 = Early childhood	−0.45	0.22	−0.88	−0.03	−2.08	0.04*	Q* = 93.65, d*f* = 3, *p* = 0.001***
	2 = Primary Educ.	−0.45	0.26	−0.97	0.07	−1.69	0.09	
	3 = Secondary Educ.	0.87	0.23	0.42	1.31	3.80	0.001***	
Educational subject	1 = ICT and Eng.	0.14	0.24	−0.33	0.61	0.60	0.55	Q* = 21.79, d*f* = 4, *p* = 0.001***
	2 = Math	−0.38	0.10	−0.58	−0.17	−3.61	0.001***	
	3 = Sciences	0.34	0.28	−0.21	0.88	1.21	0.23	
	4 = Teacher training	0.80	0.41	−0.01	1.61	1.93	0.05*	
Instruction duration	1 = 1 week ≤ dur. < 1 month	0.14	0.22	−0.30	0.57	0.60	0.55	Q* = 49.80, d*f* = 2, *p* = 0.001***
	2 = 1 semester ≤ dur. < 1 year	−1.28	0.19	−1.65	−0.90	−6.63	0.001***	
Research design	1 = Quasi-experimental	−0.58	0.25	−1.06	−0.10	−2.35	0.02*	
Sample size	1 = Large	−1.15	0.18	−1.50	−0.81	−6.56	0.001***	

### 5.8 Publication bias

**Figure 3:**
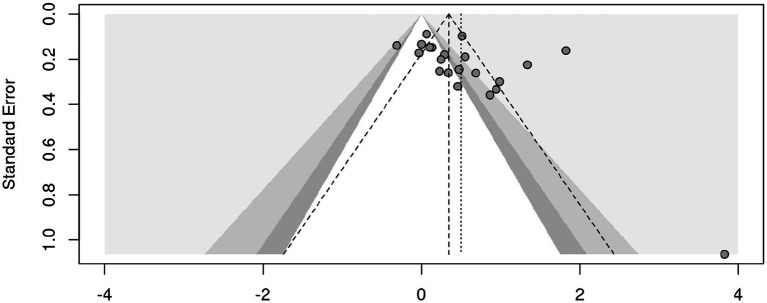
Funnel plot.

The funnel plot (Figure 3) displays the effect sizes on the *x*-axis against standard errors on the *y*-axis, with the larger studies (smaller standard errors) clustering near the top and the smaller studies (larger standard errors) spreading out towards the bottom. Further, the plot shows a slight asymmetry, with more studies skewed to the right, especially among the smaller studies at the bottom, suggesting that smaller studies reporting larger positive effects are overrepresented.

These results are confirmed by a Rank Correlation Test for Funnel Plot Asymmetry which was statistically significant and positive (Kendall’s tau: 0.4571, *p*-value: 0.0033) suggesting the presence of publication bias. Furthermore, a Regression Test for Funnel Plot Asymmetry was also statistically significant with a *z*-value of 3.3348 and a *p*-value of 0.0009. The estimated effect size as the standard error approaches zero was *b* = −0.2091, with a confidence interval of −0.6749 to 0.2567, indicating potential publication bias where smaller studies may report larger effect sizes.

To assess potential publication bias, Duval and Tweedie’s (2000) Trim-and-Fill method using the L-estimator to correct for funnel plot asymmetry was applied. The analysis was conducted in two steps: (1) identifying and removing extreme outliers, and (2) applying Trim-and-Fill to estimate and adjust for missing studies. To reduce the influence of extreme values, six outliers were identified and removed from the dataset, reducing the number of studies from *k* = 24 to *k* = 18. Studies are defined as outliers when their 95% confidence interval lies outside the 95% confidence interval of the pooled effect. Following outlier removal, Trim-and-Fill imputed three potentially missing studies on the left side of the funnel plot. After removing six outliers, the Trim-and-Fill adjusted estimate increased to *g* = 0.3181, with a narrower confidence interval [0.1672, 0.4690], which remained statistically significant (*p* < 0.0001). The funnel plot became more symmetric after outlier removal, suggesting that the initial asymmetry was at least partially driven by extreme values rather than systematic publication bias.

**Figure A1:**
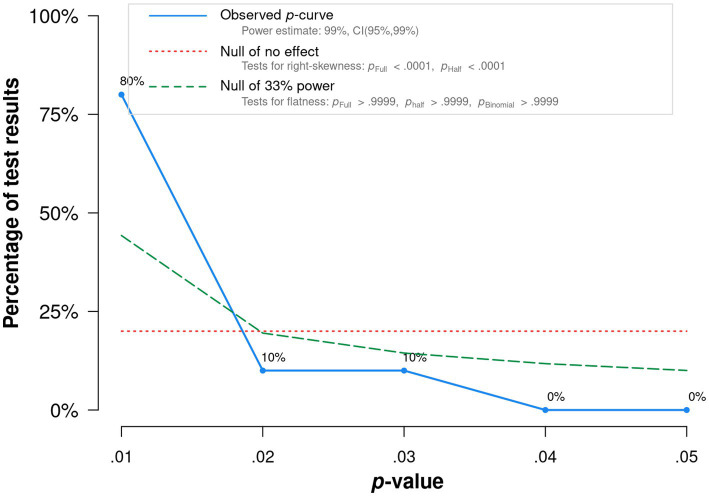
*P*-curve analysis.

On the other hand, the *p*-curve analysis (Figure A1) strongly indicates that the analyzed studies have substantial evidential value, are likely to reflect true effects, and are not just a result of selective reporting or *p*-hacking. The extremely low *p*-values for the right-skewness test and high-power estimate further reinforce this conclusion.

## 6 Discussion and conclusions

Discussions around AI are often accompanied by words like “potential,” “promise” and “hope.” While optimism is needed, it often risks overshadowing the need for tangible, measurable outcomes. It is important to not forget that AI is no stranger to hype and in fact, it has known several waves of hype and winters before. Such optimism will only be justified if the technology begins delivering real-world impact that addresses current challenges. Therefore, the present study aimed to examine in a systematic way what AI has achieved in terms of impact on students’ outcomes in BL environments through a meta-analysis of 21 studies.

### 6.1 Effect of AI on students’ learning achievement in blended learning

The present study revealed that AI has an overall medium effect size (*g* = 0.50) on learning achievement in blended learning, which is higher than the small effect found by Vo et al. (2017) when investigating the effect of blended learning without the AI component on students’ learning achievement. This implies that AI has improved students’ learning achievement in blended learning. This improvement could be attributed to the various features —and advances— provided by AI applications that can play the same role as teachers or instructors, students, or peers in blended learning (Park and Doo, 2024). AI also supports blended learning by providing personalized learning experiences and optimizing course delivery (Lee et al., 2020). Specifically, personalized systems had the highest effect on blended learning, where it achieved a large effect (*g* = 0.88), which could be explained as personalized systems could free up the teachers from the routine and repetitive tasks of preparing lesson plans and grading answer sheets. The effect of ITS on blended learning was found to be medium (*g* = 0.42). This result could be attributed to the effectiveness of ITS in a blended environment, by enabling teachers to consider a variety of models for how they might integrate ITS into their lessons to accomplish usage and blended learning guidelines (Phillips et al., 2020). However, this decrease of ITS effect compared to other AI applications (e.g., personalized learning and chatbots) reveal the importance of investigating instructional approaches used within AI applications and the effective implementation of human-machine collaboration in education.

However, it is worth noting that the effectiveness of AI in blended learning was not very high, i.e., only medium effect size, raising questions about what might hinder AI in reaching its full potential when it comes to enhancing students’ learning achievement in blended learning. This calls for more investigation in this regard to unpack both how AI might be implemented in blended learning and what might hinder its effect.

### 6.2 Educational context moderates the AI effect size in blended learning

The study found that AI had the highest effect, large (*g* = 0.70), in higher education. This could be explained by students in higher education are using AI applications for improving their problem-solving skills, conceptual understanding, and overall learning outcomes, as well as to enhance their engagement, promoting personalized learning experiences, and revolutionizing assessment strategies (Shi et al., 2023), which is greatly increasing students’ motivation to learn (Wang and Jan, 2022). However, the findings revealed that AI is less effective in early educational levels. This might be because children do not understand how AI works and the knowledge behind it. Additionally, there is not much known about how teachers can enhance children’s learning with AI applications with a methodological and appropriate approach. Moreover, there is a shortage of research on AI education for children who have no prior knowledge of computer programming and robotics. In terms of educational subjects, the current study showed that AI on blended learning has a large effect size (*g* = 0.88) in ICT and engineering. This could be explained by the potential of AI to enhance individual projects, as well as creative problem-solving abilities using technological skills such as computational thinking and data-driven reasoning. In fact, AI-generated teaching practices have proven to increase learning outcomes among engineering students (Chiang, 2021). Such knowledge suggests that AI integration strategies should be tailored to specific educational contexts in blended learning, avoiding a one-size-fits-all approach.

### 6.3 Experiment type moderates the AI effect size in blended learning

The present study revealed that the AI conducted experiment moderates the effect size on learning achievement in blended learning. Specifically, the findings indicated that AI on blended learning has a very large effect size (*g* = 1.14) on short-term interventions (1 week to 1 month). This result could be explained as this duration is considered sufficient to promote learners’ familiarization and stimulate motivation for proper engagement and achievement (Merilampi et al., 2014). It is noted that the AI effect size in long-term interventions (1 semester to 1 year) was negligible (*g* = 0.08) and this could be clarified as students lose interest and motivation in a given technology as time passes (Tlili, 2024). Among methodologists, there was significant discussion about determining the appropriate intervention duration for technological implementations. For instance, Slavin (1986) suggested longer intervention duration when using a given technology to draw solid evidence about that technology effect beyond the temporary technology novelty effect. Others, on the other hand, highlighted concerns related to that longer intervention duration might lead to a dip in performance and confidence (Cung et al., 2019). Particularly, longer AI interventions might lead to a poorer implementation fidelity, hence causing a shift from the initial experiment goals and achieving low effects (Tlili et al., 2025; Wang et al., 2023).

In terms of experiment design, the present study revealed that AI on blended learning has medium effect sizes when it is used as quasi-experimental design (*g* = 0.42) and true experimental design (*g* = 0.52). This similarity between both experiment designs can be explained with both designs being based on testing and validating hypotheses and understanding AI in multiple contexts and subjects (Ofosu-Ampong, 2024). Additionally, most experimental studies improved learning through the use of AI applications. In terms of sample size, the current study pointed out that AI on blended learning has a medium size effect (*g* = 0.57) in smaller sample sizes (≤250 participants), while the effect size was small (*g* = 0.24) in larger sample sizes (>250 participants). These results could be attributed to the nature of small sample sizes, as personalized and interactive learning approaches can be more feasible, allowing for supportive feedback and individualized support. On the other hand, large sample sizes may require more collaborative learning platforms, which can affect the depth of engagement (Tlili et al., 2024).

### 6.4 Conclusion

Through a rigorous meta-analysis of 21 studies, this research provides compelling evidence for the positive impact of AI on student learning achievement in BL environments. The findings indicate that AI-powered tools and platforms can significantly enhance learning achievement. However, it is important to acknowledge that the effectiveness of AI in BL is influenced by several factors, including the specific AI technology, the instructional design, and the characteristics of the learners. To maximize the benefits of AI, it is crucial to carefully consider these factors and implement AI-enhanced learning strategies in a thoughtful and systematic manner.

Future research should explore the long-term impacts of AI on student learning, particularly in terms of critical thinking, problem-solving, and creativity, as well as the optimal integration of AI tools into various learning contexts. Moreover, study designs aimed at assessing learning outcomes (such as pre-posttest, experimental, or quasi-experimental settings) focus on taking snapshots of students’ learning before and after a specific intervention (e.g., using AI). Research that focuses on the process rather than only on the outcomes is needed to understand whether students are making use of AI in an effective way.

There is paucity of studies in underperforming contexts (e.g., primary education) and evidence from these areas is rather thin. Additionally, there is an ongoing tension about whether AI should be used in primary education or not. For instance, the President von der Leyen stated in the State of the European Union 2025 “I strongly believe that parents, not algorithms, should be raising our children.” Nevertheless, research is needed to examine such context given the potential benefits of AI, the evidence from other contexts and the fact that AI is expected to be an important player in everyday life. It is crucial to understand if and how AI can enhance learning in underperforming primary education settings, address challenges like teacher readiness, infrastructure, and safety. Research should address if and to what extent AI can enhance students’ engagement, support and personalization. Such research is needed to optimize learning and most importantly to inform evidence-based strategies for integrating AI effectively into the classrooms. The same applies to other areas and disciplines where AI has been rarely applied. However, extending AI to schools requires digital infrastructure, teacher training, and considering ethical considerations. Some schools lack stable internet access, adequate devices, and most importantly, the technical and practical expertise to effectively and safely integrate AI. Furthermore, AI must be implemented in ways that uphold data privacy, avoid biases that could further disadvantage certain student groups.

The present meta-analysis demonstrates AI’s potential to improve learning achievement in blended environments, particularly through personalized and adaptive functionalities. This potential is highly synergistic with inclusive education goals by enabling unprecedented levels of differentiation, accessibility support, and early intervention tailored to individual learner needs. It also highlights critical moderating variables that determine whether this potential AI efficacy translates into genuinely inclusive benefits. Ultimately, AI in blended learning can be a powerful lever for inclusive education, but only if its deployment is intentionally designed, critically evaluated, and continuously refined with equity and human agency at its core.

### 6.5 Future research

The agenda for future research on AI can be long given that AI is extending to all areas of research and practice with little research on its long-term impact. While the present study has shown primary evidence of the impact of AI in some contexts, research is needed to address areas where evidence is lacking like early school years. Most importantly, the knowledge about the long-term impact of AI is still limited, requiring longitudinal studies that track its effects over time. Research should explore how AI influences cognitive development, learning outcomes, and social interactions in young children. Furthermore, it is important to investigate how AI affects students’ motivation, dependence on technology and students’ soft skills. Future research should also investigate the unintended consequences of AI deployment, such as bias, misinformation, and environmental sustainability. Alignment between AI and students’ needs has never been more important to ensure that AI-driven educational solutions support rather than hinder student development. Unfortunately, research on alignment is lacking and is therefore badly needed.

Finally, blended learning has always meant blending online and face-to-face learning. With the emergence of AI, it may expand beyond this traditional definition, introducing a dynamic interaction between human teachers, students, and intelligent systems. However, this shift raises critical questions about the evolving role of teachers in AI-enhanced classrooms which calls for research on safe implementations as well as monitoring of impact.

### 6.6 Limitations

While the reliability of this meta-analysis has been investigated through several methods, including bias assessment, it still has several limitations that should be acknowledged. For instance, the findings of the present study might be limited by the search keywords or databases. Additionally, it covered only journal papers written in English. Therefore, future researchers could complement this study by including papers written in other languages as well as covering more electronic databases and search keywords.

## Data Availability

The original contributions presented in the study are included in the article/supplementary material, further inquiries can be directed to the corresponding author/s.
